# Assessment of Fractional Flow Reserve from Coronary CT Angiography Using a Deep Learning-Based Algorithm: A Multicenter Retrospective Study

**DOI:** 10.3390/diagnostics16050762

**Published:** 2026-03-04

**Authors:** Ludovica R. M. Lanzafame, Claudia Gulli, Maria Teresa Cannizzaro, Bruno Francaviglia, Laura M. Chisari, Leon D. Grünewald, Vitali Koch, Christian Booz, Thomas J. Vogl, Luca Saba, Silvio Mazziotti, Tommaso D’Angelo

**Affiliations:** 1 Diagnostic and Interventional Radiology Unit, BIOMORF Department, University Hospital “Policlinico G. Martino”, Via Consolare Valeria 1, 98100 Messina, Italy; 2 Radiology UOSD CAST, Universitary Hospital A.O.U. Policlinico “G.Rodolico-San Marco”, 95123 Catania, Italy; 3 Division of Cardiology, Policlinico Hospital, University of Catania, 95123 Catania, Italy; 4 Department of Diagnostic and Interventional Radiology, University Hospital Frankfurt, Theodor-Stern-Kai 7, 60590 Frankfurt am Main, Germany; 5 Division of Experimental Imaging, Department of Diagnostic and Interventional Radiology, University Hospital Frankfurt, Theodor-Stern-Kai 7, 60590 Frankfurt am Main, Germany; 6 Department of Radiology, University of Cagliari, 09045 Cagliari, Italy

**Keywords:** Artificial Intelligence, deep learning, coronary artery disease, coronary stenosis, coronary computed tomography angiography, invasive coronary angiography, fractional flow reserve

## Abstract

**Objectives:** To assess the diagnostic accuracy of a deep learning (DL)-based algorithm for non-invasive computation of fractional flow reserve (FFR-CT) from coronary computed tomography angiography (CCTA) and to evaluate the model’s ability to automatically assign cardiovascular risk categories according to the Coronary Artery Disease–Reporting and Data System (CAD-RADS). **Materials and Methods:** Sixty patients with suspected coronary artery disease who underwent both CCTA and invasive coronary angiography (ICA) were retrospectively included in this multicenter study. Curved multiplanar reconstructions derived from CCTA were analyzed by the deep learning-based model to estimate FFR-CT values and to automatically assign CAD-RADS risk categories. The diagnostic performance of the software for the identification of hemodynamically significant coronary stenoses was evaluated using ICA as the reference standard. Receiver operating characteristic (ROC) curve analysis was performed to determine the area under the curve (AUC), sensitivity, and specificity on both a per-patient and per-vessel basis. Finally, agreement between CAD-RADS risk categories assigned by the DL algorithm and those determined by an expert radiologist was assessed. **Results:** FFR-CT demonstrated high diagnostic accuracy, with AUC of 0.935, sensitivity of 93.2%, specificity of 93.7%, and excellent agreement with reference standard (k = 0.836) on a per-patient level. Per-vessel diagnostic performance was consistently high across all major coronary arteries, with the left anterior descending artery (LAD) showing the highest accuracy (AUC = 0.932). Automated CAD-RADS classifications generated by the software showed good agreement with those assigned by human (k = 0.765). **Conclusions:** The DL-based model demonstrated high diagnostic accuracy and represents a promising noninvasive approach for ischemia assessment and cardiovascular risk stratification.

## 1. Introduction

Coronary computed tomography angiography (CCTA) has emerged as a cornerstone in the non-invasive evaluation of patients with moderate clinical pre-test likelihood of coronary artery disease (CAD), due to its high sensitivity and strong negative predictive value in identifying coronary stenoses [1]. However, its ability to predict lesion-specific ischemia remains limited, especially in patients with moderate stenoses (typically defined as 50–69% luminal narrowing) [2]. This often leads to referral for invasive coronary angiography (ICA) and pressure-wire assessments, such as fractional flow reserve (FFR) or instantaneous wave-free ratio (iFR), to further clarify the presence of hemodynamically significant disease [3,4]. Artificial intelligence (AI) has been increasingly applied to automated image analysis tasks, with the potential to streamline image interpretation workflows and facilitate timely and efficient reporting in clinical practice [5,6]. AI-based tools are reshaping radiology by enhancing image analysis, automating detection and segmentation tasks, and improving diagnostic accuracy and workflow efficiency. It enables the extraction of quantitative imaging features and supports more consistent and reproducible interpretations [5]. Importantly, AI-driven reconstruction and optimization techniques also allow reduction in radiation dose while preserving diagnostic image quality [7,8]. Recently, deep learning (DL)-based models have been developed to rapidly predict lesion-specific ischemia directly computing fractional flow reserve from CCTA datasets (FFR-CT) [9,10]. At present, the dissemination of AI-based tools remains largely confined to large, often university-affiliated centers, primarily due to their high costs and infrastructural requirements. However, emerging collaborative and system-level approaches suggest that wider implementation may become increasingly feasible over time, potentially enabling broader access beyond high-resource institutions [11].

The aim of the study was to assess the diagnostic performance of a commercially available DL-based FFR-CT algorithm for the detection of hemodynamically significant coronary stenoses, using ICA as reference standard, and to evaluate the software’s capability to automatically assign Coronary Artery Disease–Reporting and Data System (CAD-RADS) categories.

## 2. Materials and Methods

Institutional Review Board approval was obtained, with a waiver of informed consent due to the retrospective, observational design.

### 2.1. Patient Population

**Figure 1:**
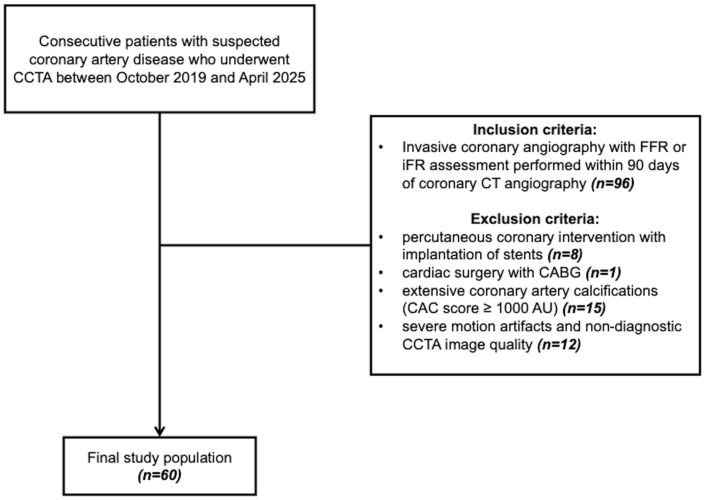
Study flowchart.

This multicenter retrospective study was conducted at two different university hospitals and included consecutive patients with suspected CAD who underwent both CCTA and ICA within a 90-day interval between October 2019 and April 2025. Exclusion criteria included: (i) history of percutaneous coronary intervention with implantation of stents; (ii) history of cardiac surgery with coronary artery bypass grafting (CABG); (iii) extensive coronary artery calcifications (CAC score ≥ 1000 AU); (iv) severe motion artifacts and non-diagnostic CCTA image quality. The flowchart illustrating the patient selection process is shown in Figure 1.

### 2.2. Acquisition Protocol and Image Reconstruction

CCTA examinations were performed using a 64-row dual-layer CT scanner (IQon CT, Philips Healthcare, Best, The Netherlands) at Center 1 (“G. Martino”, Messina University Hospital) and a 320-row Toshiba Aquilion ONE (Toshiba Medical Systems Corporation, Otawara, Japan) at Center 2 (“G.Rodolico-San Marco”, Catania University Hospital). All examinations were performed with the patient in the supine position, and imaging was obtained in the cranio-caudal direction. Firstly, a prospective ECG-gated non-contrast scan (120 kVp tube voltage, 40 mAs tube current) was acquired for the assessment of coronary artery calcium (CAC) score. Subsequently, a contrast-enhanced scan was performed using a retrospective ECG-gated protocol. Intravenous contrast (Iomeprol 400, Iomeron, Bracco, Milan, Italy) was administered at 0.85 mL/kg injected at 5 mL/s, followed by a 50 mL saline flush. Automated bolus tracking was used, with a region of interest placed in the descending aorta to trigger image acquisition. The tube voltage was set at 120 kVp, with tube current modulation based on patient body weight. Common acquisition parameters included a matrix size of 512 × 512 and a field of view of 220–250 mm. Gantry rotation time and detector configuration varied between the two institutions, being 0.27 s and 64 × 0.625 mm at Center 1 and 0.35 s and 320 × 0.5 mm at Center 2, respectively. Before acquisition, all patients received premedication with sublingual vasodilator (isosorbide dinitrate), when not contraindicated; intravenous β-blocker was administered to patients with a heart rate ≥ 70 bpm (esmolol hydrochloride). CCTA images were reconstructed along the R–R interval, during end-diastolic phases or those providing the best image quality. Image reconstruction was performed using a slice thickness of 0.67 mm with a reconstruction increment of 0.34 mm on the IQon CT system, and a slice thickness of 0.5 mm with a reconstruction increment of 0.25 mm on the Aquilion ONE system. The acquisition parameters at both centers are summarized in Table 1.

### 2.3. Deep Learning Based Analysis

CorEx version 2.4.1 (Spimed-AI, Paris, France) was used for both FFR-CT computation and CAD-RADS classification. The software is a semi-automated DL-based model, trained on the Inception-v3 convolutional neural network architecture and developed for the automated interpretation of CCTA images [12,13,14,15].

Curved multiplanar reconstructions (cMPRs) from CCTA datasets acquired at both centers were generated at Center 1 by a radiologist with 4-years of expertise (C.G.) in cardiovascular imaging, during this phase, the same reader was responsible for excluding datasets deemed to have insufficient image quality. Reconstructions were performed on a workstation using dedicated software (IntelliSpace Portal, version 8.0, Philips Healthcare, Best, The Netherlands). cMPRs were reconstructed at 40° angular intervals to cover the full circumference of each major coronary artery. For each patient, a total of 27 curved MPR images were generated, including nine for the left anterior descending (LAD), nine for the right coronary artery (RCA), and nine for the circumflex artery (LCx). The left main coronary artery was included in the reformatted images of both LAD and LCx. Coronary artery centerlines were manually reviewed and corrected, when necessary, by the same radiologist. The resulting cMPR datasets were subsequently uploaded to the CorEx platform and automatically processed by the DL-based algorithm to generate binary FFR-CT predictions (≤0.80 vs. >0.80) and automated CAD-RADS classifications.

### 2.4. Reference Standard

ICA data, including FFR and iFR measurements, were collected from the patients’ clinical records at each participating institution and recorded for analysis. CCTA datasets were independently reviewed by two board-certified radiologists with expertise in cardiovascular imaging (T.D. and M.T.C.), who were blinded to ICA results, using axial images and cMPRs on locally available dedicated workstations with vendor-specific software at each institution. For each patient, CAD severity was categorized according to the Coronary Artery Disease–Reporting and Data System 2.0 as follows: CAD-RADS 0, no plaque or stenosis; CAD-RADS 1, minimal stenosis (1–24%); CAD-RADS 2, mild stenosis (25–49%); CAD-RADS 3, moderate stenosis (50–69%); CAD-RADS 4, including both CAD-RADS 4A, severe stenosis (70–99%) and CAD-RADS 4B, left main stenosis ≥50% or three-vessel disease with ≥70% stenosis; and CAD-RADS 5, a total occlusion (100%) [16].

### 2.5. Statistical Analysis

Statistical analysis was performed using a commercially available software (MedCalc Software, version 20.1, Ostend, Belgium). Data distribution was assessed with the Shapiro–Wilk test. Categorical variables were indicated as numbers and percentages. Normally distributed data are expressed as mean ± standard deviation (SD), whereas non-normally distributed data are reported as median and interquartile range [IQR]. Comparisons between groups were performed using a two-tailed *t*-test for normally distributed data, and the Wilcoxon test for non-normally distributed data.

The diagnostic accuracy of the DL-based FFR-CT software was assessed on both a per-patient and per-vessel basis, using invasive FFR and iFR as reference standards, with vessel-specific ischemia defined by thresholds of FFR ≤ 0.80 and iFR ≤ 0.89 [3,17,18,19]. To this end, receiver operating characteristic (ROC) curves were constructed with assessment of the area under the curve (AUC). Accuracy, sensitivity, specificity, positive predictive value (PPV) and negative predictive value (NPV) were also assessed. Agreement between FFR-CT and invasive measurement was further evaluated using Cohen’s kappa coefficient (k).

Finally, weighted Cohen’s kappa was calculated to assess the level of agreement between CAD-RADS scores assigned by expert readers at each institution and those automatically generated by the AI-based software.

Results were interpreted as follows: slight or poor agreement (k < 0.20), fair agreement (k = 0.20–0.40), moderate agreement (k = 0.40–0.60), good agreement (k = 0.60–0.80), and excellent agreement (k > 0.80).

## 3. Results

### 3.1. Patient Population

From an initial cohort of 96 patients, 36 were excluded according to the following criteria: (i) history of percutaneous coronary intervention with implantation of stents (*n* = 8); (ii) history of cardiac surgery with CABG (*n* = 1); (iii) extensive coronary artery calcifications (CAC score ≥ 1000 Agatston units) (*n* = 15); (iv) severe motion artifacts and non-diagnostic CCTA image quality (*n* = 12).

The final patient population included 60 patients (45 males and 15 females; mean age 69.5 ± 9.6), 34 from Center 1 and 26 from Center 2. The median interval days between CCTA and ICA was 33.0 [10.0–73.5].

FFR measurements were available in 37 of 60 patients (61.7%), with 54 out of 180 vessels (30%) classified as positive; iFR measurements were available in 23 out of 60 patients (38%), with 14 of 180 vessels (8%) classified as positive. No patient underwent both FFR and iFR assessment. Patient demographic characteristics are reported in Table 2.

### 3.2. Diagnostic Performance of FFR-CT

On a per-patient basis, the DL-based software demonstrated high diagnostic performance, showing an AUC of 0.935 (95% CI: 0.840–0.982). Sensitivity, specificity PPV, NPV and accuracy were 93.2% (95% CI: 81.3–98.6%), 93.7% (95% CI: 69.8–99.8%), 97.6% (95% CI: 85.9–99.6%), 83.3% (95% CI: 62.5–93.7%) and 93.3% (95% CI: 83.8–98.1%), respectively. Excellent agreement was observed between FFR-CT and the invasive reference standard (k = 0.836).

A total of 180 vessels were analyzed, with 60 vessels for each of the three main coronary arteries. The AUC was 0.902 (95% CI: 0.849–0.941). Sensitivity and specificity were 84.5% (95% CI: 72.6–92.6%) and 95.9% (95% CI: 90.7–98.7%), respectively. PPV, NPV and accuracy were as follows: 90.7% (95% CI: 80.5–95.8%), 92.8% (95% CI: 87.7–95.9%) and 92.2% (95% CI: 87.3–95.7%).

**Figure 2:**
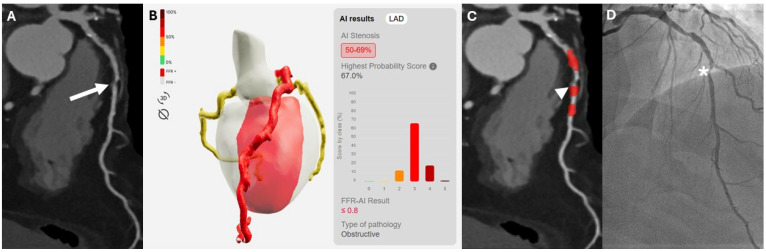
A 72-year-old female patient with a moderate (50–69%) stenosis in the mid segment of the left anterior descending artery (LAD), identified on curved multiplanar reconstructions (cMPR) ((**A**)—white arrow), classified as hemodynamically significant by the deep learning-based model (**B**) as indicated by the red heatmap ((**C**)—white arrowhead) and subsequently confirmed by invasive coronary angiography ((**D**)—asterisk).

**Figure 3:**
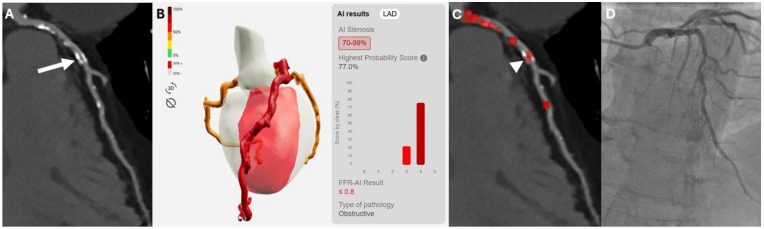
Exemplary false-positive case in a 75-year-old male patient. The deep learning-based model (**B**) identified a severe, hemodynamically significant stenosis, in the proximal segment of the left anterior descending artery (LAD), indicated by a red heatmap ((**C**)—white arrowhead) and associated with FFR-CT values ≤ 0.8. Curved multiplanar reconstruction (cMPR) images (**A**) demonstrated a moderate (50–69%) calcified stenosis (white arrow), which was confirmed as non-ischemic by invasive coronary angiography, with measured FFR > 0.8 (**D**).

Excellent agreement was also observed on a vessel-based analysis (k = 0.819). In the exploratory subgroup analysis of vessels with moderate stenoses (24/180; 13.3%), invasive coronary angiography demonstrated significant coronary stenoses in 11/24 cases, among these the algorithm correctly detected ischemic lesions in 72.7% of cases (8/11). The remaining moderate stenoses that were not associated with hemodynamically significant disease were correctly classified as such in 12 out of 13 cases (92.3%). The diagnostic accuracy in this subgroup of patients showed an AUC was 0.825 (95% CI: 0.617–0.948), whereas sensitivity, specificity, PPV, NPV and accuracy were as follows: 72.7% (95% CI: 39.1–93.9%), 92.3% (95% CI: 63.9–99.8%), 88.9% (95% CI: 54.0–98.1%), 80.0% (95% CI: 62.6–95.3%), and 83.3% (95% CI: 62.6–95.3%). Two cases of moderate coronary stenosis are illustrated in Figure 2 and Figure 3, representing a true-positive and a false-positive classification, respectively.

A vessel-specific sub-analysis, performed to evaluate the diagnostic performance of the software for each major coronary artery, showed consistently strong diagnostic performance across all major coronary arteries, with LAD exhibiting the highest accuracy. The algorithm achieved an AUC of 0.932 (95% CI: 0.836–0.981%), sensitivity of 94.1% (95% CI: 80.3–99.3%), specificity of 92.3% (95% CI: 74.9–99.0%), and an accuracy of 93.3% (95% CI: 83.8–98.1%). The PPV and NPV were 94.1% (95% CI: 80.8–98.4%) and 92.3% (95% CI: 75.7–97.9%), respectively. Comparable diagnostic performance was also observed across the remaining epicardial coronary vessels, with detailed results summarized in Table 3.

**Figure 4:**
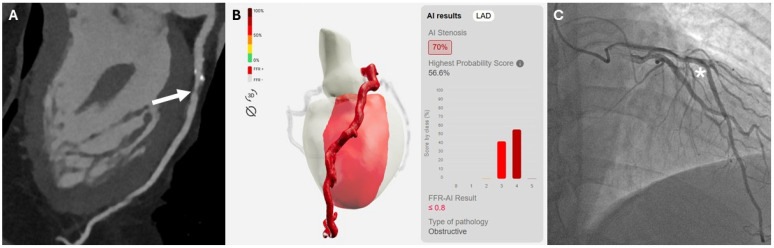
A 64-year-old male patient with suspected coronary artery disease. Curved multiplanar reconstruction (cMPR) from coronary computed tomography angiography demonstrated a mixed atherosclerotic plaque in the proximal segment of the left anterior descending artery (LAD) ((**A**)—white arrow). Analysis of the cMPR images by the deep learning-based model identified a hemodynamically significant lesion with FFR-CT values ≤ 0.80 (**B**). Invasive coronary angiography confirmed the presence of a significant stenosis in the proximal LAD ((**C**)—asterisk).

**Figure 5:**
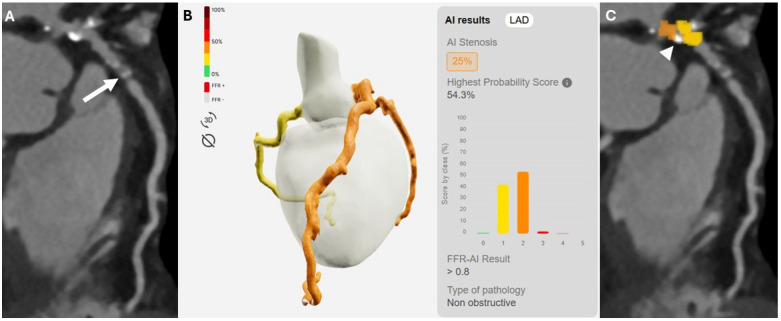
Curved multiplanar reconstruction (cMPR) images (**A**) from a 78-year-old female patient demonstrating a severe stenosis in the mid segment of the left anterior descending artery (LAD) caused by a non-calcified plaque (white arrow) and classified as CAD-RADS 4, which was subsequently confirmed by invasive coronary angiography. Analysis by the deep learning-based software (**B**) identified only non–hemodynamically significant lesions, highlighted by a yellow–orange heatmap overlay ((**C**)—white arrowhead), underestimating lesion severity and resulting in a false-negative classification.

Figure 4 and Figure 5 illustrate a true-positive and a false-negative case of left anterior descending artery (LAD) stenosis, respectively.

### 3.3. CAD-RADS Agreement

Automated cardiovascular risk stratification yielded the following CAD-RADS distribution: CAD-RADS 0 in 0/60 patients (0%); CAD-RADS 1 in 3/60 patients (5%), CAD-RADS 2 in 11/60 (18.3%), CAD-RADS 3 in 7/60 (11.7%), CAD-RADS 4 in 28/60 (46.7%), and CAD-RADS 5 in 11/60 patients (18.3%).

No statistically significant differences were observed between CAD-RADS scores assigned by expert readers and those automatically generated by the DL-based software (*p* = 0.567), with good agreement between the two methods (k = 0.765).

## 4. Discussion

The purpose of this study was to demonstrate the diagnostic performance of a DL-based FFR-CT algorithm in identifying vessel-specific ischemia using FFR and iFR as reference standard, and to evaluate the automated model’s CAD-RADS prediction. Both per-patient and per-vessel analyses showed high sensitivity, specificity, and AUC values for the identification of hemodynamically significant coronary stenoses based on FFR-CT.

The observed results are consistent with prior DL-based FFR-CT studies which have demonstrated per-patient AUCs in the range of 0.86–0.92 [9,20,21]. These findings demonstrated the model’s reliability in both global patient evaluation and vessel-specific ischemia assessment.

Exploratory subgroup analysis by coronary territory consistently revealed consistent performance across LAD, LCx, and RCA vessels. The LAD territory had the highest diagnostic metrics, with an AUC of 0.932 and sensitivity and specificity both exceeding 90%. In contrast, the LCx territory showed lower sensitivity (70%) and agreement (k = 0.687). The LCx typically presents with a smaller vessel caliber and increased tortuosity compared with the LAD, which may complicate accurate image reconstruction and lumen assessment on CCTA [9,22]. The RCA assessment also demonstrated solid performance (AUC = 0.846), despite slight reductions in sensitivity compared to LAD, as reported in similar FFR-CT studies [20,23]. Specifically, the RCA is typically surrounded by a greater amount of pericoronary adipose tissue compared with the LAD, exhibits higher mobility, and is particularly susceptible to motion artifacts due to its more perpendicular movement relative to the CT acquisition plane. This combination of anatomical and technical factors may hinder optimal cMPR quality [24,25]. Collectively, these findings suggest that vessel-specific anatomical characteristics may contribute to the variable accuracy observed in DL-based functional assessment.

A particularly noteworthy result was the model’s diagnostic accuracy in borderline stenoses (50–69%), where conventional CCTA alone often lacks specificity. The relatively small sample size in this category likely contributed to lower sensitivity estimates and wider confidence intervals, thereby limiting the precision of these findings; nevertheless, the algorithm correctly identified ischemic and non-ischemic lesions in a substantial proportion of cases, holding good promise in ambiguous clinical scenarios. This aligns with emerging data highlighting the utility of DL-based FFR-CT tools in refining decision-making for intermediate lesions, where further invasive investigations are frequently required for clarification [20,26]. The analysis of discordant cases also provided additional insight into the model’s limitations. Among the false negatives (9/180 vessels), most were associated with low image quality and suboptimal vessel opacification, reflecting the potential impact of technical factors such as signal-to-noise/contrast-to-noise ratio and motion artifacts on AI-based models, while nonetheless preserving superior diagnostic performance compared with the assessment of coronary stenosis by CT alone [9,27]. Additionally, models based on uploaded cMPRs may not detect ischemia caused by microvascular dysfunction, as downstream resistance cannot be quantified by CCTA-derived algorithms [10]. On the other hand, despite the exclusion of patients with a very high coronary calcium burden (CAC > 1000), false positives (5/180 vessels) were frequently linked to heavy calcifications. In these cases, blooming artifacts likely led to overestimation of stenosis severity and consequent misclassification of functional significance [20,23,28,29]. Other technical factors, such as motion artifacts may also negatively influence diagnostic performance. However, in the present study, datasets with inadequate image quality were excluded a priori to ensure reliable analysis. Future investigations specifically addressing more challenging imaging conditions will be important to further validate the robustness of the software in real-world, heterogeneous scenarios.

Finally, the DL-based FFR-CT model showed good concordance with human-assigned CAD-RADS scores (k = 0.765), reinforcing its utility not only in ischemia detection but also in structured risk stratification, consistent with previous studies on the use of AI in CCTA. However, despite the overall good agreement, a non-negligible rate of misclassification was observed. This aspect should not be underestimated, particularly in cases of risk-class downstaging, which may have significant implications for subsequent patient management. Therefore, at present, human oversight remains essential to ensure appropriate clinical interpretation and decision-making. Furthermore, in our dataset, no patients were classified as CAD-RADS 0, reflecting a cohort characterized by a relatively high cardiovascular risk profile, with the majority of patients falling into category 4. This distribution is consistent with the higher likelihood of referral to invasive procedures in patients belonging to these higher-risk categories.

Medical AI-based approaches are transitioning from the research setting to routine clinical practice and, with the growing number of CCTA examinations, automated CAD-RADS assignment could further streamline reporting, especially in high-volume centers [10,12,30,31,32]. In this setting, a tool capable of delivering automated CAD-RADS classification within a few minutes, while simultaneously providing information on the hemodynamic significance of stenoses, may contribute to a more efficient and integrated clinical workflow.

This study has several limitations. Firstly, its retrospective design and the modest sample size, along with the incomplete availability of detailed baseline comorbidity data, may limit the generalizability of the findings and may have had a more pronounced impact on statistical power in subgroup analyses, particularly those focusing on moderate-grade stenoses and vessel-specific assessments of individual coronary arteries. Larger, prospective, multicenter studies and different healthcare settings are needed to further validate the performance of the DL-based model. Moreover, the relatively long interval between CCTA and ICA may introduce temporal bias related to possible changes in coronary disease status over time, potentially resulting in discrepancies in the assessment of disease severity. However, in routine clinical practice, the timing of invasive angiography often varies due to logistical factors, including specialist availability and patient-related considerations. Although longitudinal studies have shown that FFR may gradually decrease over long-term follow-up in patients with stable coronary artery disease, short-term variations over a period of weeks are generally minimal in the absence of acute events. Therefore, the interval between CCTA and ICA in our cohort is unlikely to have significantly influenced the observed FFR correlation [33]. Secondly, the radiologist’s manual correction of vessel centerlines significantly influenced results thus introducing operator dependency. In the present study, semi-automated centerline correction was performed by a single operator; consequently, operator dependency could not be formally assessed. In addition, CAD-RADS categorization was assigned by a single reader at each center, precluding a formal evaluation of inter-reader agreement and potentially introducing variability in category assignment. Further studies involving multiple readers, ideally with different levels of experience, are warranted to better evaluate inter-operator variability and reproducibility. A third limitation is the use of two different CT scanner models with slight variations in acquisition protocols; however, previous studies have demonstrated stable diagnostic performance of the algorithm across different scanner types and acquisition settings [30]. Finally, the use of two different indices (FFR and iFR) as reference standards may have introduced a degree of heterogeneity in the results, although the literature has consistently demonstrated a strong correlation and high diagnostic agreement between FFR and iFR in the assessment of functionally significant coronary stenoses [17,34].

## 5. Conclusions

In conclusion, the DL-based FFR-CT algorithm demonstrated promising diagnostic accuracy in identifying hemodynamically significant coronary stenoses, consistently maintaining good diagnostic performance across all major coronary arteries and showing substantial concordance with invasive measurements. The findings of this exploratory study further validate the clinical value of FFR-CT as a reliable noninvasive tool for the assessment of both anatomical and hemodynamic characteristics of coronary lesions. The integration of DL-based FFR-CT into routine clinical practice may allow functional assessment of coronary stenoses directly from CCTA images, potentially reducing the need for invasive procedures, shortening diagnostic timelines, lowering healthcare costs, and benefiting patients by avoiding additional exposure to ionizing radiation associated with ICA. Furthermore, automated CAD-RADS categorization may substantially streamline the reporting process while promoting greater standardization of structured reports, including systematic incorporation of risk categories that are not consistently reported in routine clinical practice.

## Figures and Tables

**Table 1:** Acquisition parameters for coronary artery calcium scoring and CCTA protocols across the two participating centers.

		Center 1	Center 2
**Scanner model**		IQon CT, Philips	Aquilion ONE, Toshiba
**Number of detectors (row)**		64, dual layer	320
**Gantry rotation time (s)**		0.27	0.35
**Detector configuration**		64 × 0.625 mm	320 × 0.5 mm
**CAC**	Acquisition	Prospective	
	Tube voltage (kVp)	120	
	Tube current (mAs)	40	
**CCTA**	Acquisition	Retrospective/Prospective	
	Tube voltage (kVp)	120	
	Tube current (mAs)	Weight-based	
	Scan trigger mode	Automated bolus tracking	
	Matrix size	512 × 512	
	FOV (mm)	220–250	
	Slice thickness (mm)	0.67	0.5
	Increment (mm)	0.34	0.25

CAC: Coronary Artery Calcium; CCTA: Coronary Computed Tomography Angiography; FOV: Field of View.

**Table 2:** Demographic characteristics of the study population.

Patient Population	
**Age (years)**	69.5 ± 9.6
**Males**	45/60 (75%)
**Females**	15/60 (25%)
**Center 1**	34/60 (56.7%)
**Center 2**	26/60 (43.3%)
**FFR**	37/60 (61.7%)
**iFR**	23/60 (38.3%)

FFR: Fractional Flow Reserve; iFR: Instantaneous wave-free ratio. Values are reported as mean ± SD or numbers and percentages (%).

**Table 3:** Diagnostic performance of the deep learning-based FFR-CT.

FFR-CT Diagnostic Performance						
	AUC	Sensitivity	Specificity	PPV	NPV	Accuracy
**Per-patient**	0.935 (0.840–0.982)	93.2 (81.3–98.6)	93.7 (69.8–99.8)	97.6 (85.9–99.6)	83.3 (62.5–93.7)	93.3 (83.8–98.1)
**Per-vessel**	0.902 (0.849–0.941)	84.5 (72.6–92.6)	95.9 (90.7–98.7)	90.7 (80.5–95.8)	92.8 (87.7–95.9)	92.2 (87.3–95.7)
**LAD**	0.932 (0.836–0.981)	94.1 (80.3–99.3)	92.3 (74.9–99.0)	94.1 (80.8–98.4)	92.3 (75.7–97.9)	93.3 (83.8–98.1)
**LCx**	0.830 (0.711–0.915)	70.0 (34.7–93.3)	96.0 (86.3–99.5)	77.8 (45.9–93.5)	94.1 (86.1–97.6)	91.7 (81.6–97.2)
**RCA**	0.846 (0.730–0.926)	71.4 (41.9–91.6)	97.8 (88.5–99.9)	90.9 (58.3–98.6)	91.8 (83.1–96.3)	91.7 (81.6–97.2)

AUC: Area Under the Curve; LAD: Left Anterior Descending Artery; LCx: Left Circumflex Artery; NPV: Negative Predictive Value; PPV: Positive Predictive Value; RCA: Right Coronary Artery. Results are expressed with corresponding 95% confidence intervals in brackets.

## Data Availability

The data presented in this study are available on request from the corresponding author.

## Abbreviations

The following abbreviations are used in this manuscript:

AI	Artificial Intelligence
AU	Agatston Units
AUC	Area Under The Curve
CABG	Coronary Artery Bypass Grafting
CAC	Coronary Artery Calcium
CAD	Coronary Artery Disease
CAD-RADS	Coronary Artery Disease-Reporting and Data System
CCTA	Coronary Computed Tomography Angiography
CI	Confidence Interval
CT	Computed Tomography
cMPR	Curved Multiplanar Reconstruction
DL	Deep Learning
FFR	Fractional Flow Reserve
FFR-CT	Coronary Computed Tomography Angiography-derived Fractional Flow Reserve
ICA	Invasive Coronary Angiography
IQR	Interquartile Range
iFR	Instantaneous Wave-free Ratio
LAD	Left Anterior Descending Artery
LCx	Left Circumflex Artery
NPV	Negative Predictive Value
PPV	Positive Predictive Value
RCA	Right Coronary Artery
ROC	Receiver Operating Characteristic
SD	Standard Deviation
